# Supplementary material for: PIPI-C: A Combinatorial Optimization Framework for Identifying Post-translational Modification Hot-spots in Mass Spectrometry Data
Source: Mol Cell Proteomics. 2025 Dec 23;25(2):101494. doi: 10.1016/j.mcpro.2025.101494 (PMC12834927; doi:10.1016/j.mcpro.2025.101494)
Supplement: [Supplemental Table S1] [file mmc1.pdf]

Supplementary Information

for

PIPI-C: A combinatorial optimization framework for identifying post-translational modification hot-spots in mass spectrometry data

Shengzhi Lai[1,†], Shuaijian Dai[2,†], Peize Zhao[3], Chen Zhou[1], Ning Li[4,*] and Weichuan Yu[1,*]

[1] Department of Electronic and Computer Engineering,

The Hong Kong University of Science and Technology, Hong Kong, China

[2]Department of Biomedical Engineering, School of Basic Medical Sciences,

Central South University, Hunan 410013, China

[3] Interdisciplinary Programs Office,

The Hong Kong University of Science and Technology, Hong Kong, China

[4] Ningbo No.2 Hospital, China, Zhejiang 315010, China

[†] Co-first authors

[*] Correspondence: N.L. (boningli@ust.hk, Telephone: (852) 2358-7335) and W.Y. (eeyu@ust.hk, Telephone: (852) 2358-7054)

Contents

Contents S2

1 Supplementary Note 1: Generation of simulated data sets S4

Table S1. Parameters, PTMs, and sites used in AlphaPeptDeep for simulated data sets S4

2 Supplementary Note 2: Data preprocessing and tag extraction S5

3 Supplementary Note 3: Software and parameters used in the experiments S6

Table S2. Versions of software programs used in this work S6

Table S3. Common parameters used in the experiments. A single asterisk (*) denotes the search engine incorporating the corresponding variable modification setting. Double asterisk (**) denotes the search engine with both Phe→Cys@F and Unknown:248@N-term as variable modifications. S7

4 Supplementary Note 4: Details of the 21 synthetic data sets and the results S8

Table S4. Information of the 21 synthetic data sets. Each data set contains tens of thousands of MS2 spectra with up to one PTM. S8

Figure S1. Comparison results of the synthetic data sets 01 to 07 at FDR of 0.01: bar chart, PSM numbers; pie chart, PTM numbers. S9

Figure S2. Comparison results of the synthetic data sets 08 to 14 at FDR of 0.01: bar chart, PSM numbers; pie chart, PTM numbers. S9

Figure S3. Comparison results of the synthetic data sets 15 to 21 at FDR of 0.01: bar chart, PSM numbers; pie chart, PTM numbers. S10

5 Supplementary Note 5: Results of replicated soybean data set S11

Figure S4. Replicate R02. Intersections of PSMs with fully dimethyl-labeled peptides identified from the soybean data sets. D1, D2: two samples in the data set. S11

Figure S5. Replicate R03. Intersections of PSMs with fully dimethyl-labeled peptides identified from the soybean data sets. D1, D2: two samples in the data set. S12

Figure S6. Replicate R04. Intersections of PSMs with fully dimethyl-labeled peptides identified from the soybean data sets. D1, D2: two samples in the data set. S12

Figure S7. Replicate R05. Intersections of PSMs with fully dimethyl-labeled peptides identified from the soybean data sets. D1, D2: two samples in the data set. S13

Figure S8. Replicate R06. Intersections of PSMs with fully dimethyl-labeled peptides identified from the soybean data sets. D1, D2: two samples in the data set. S13

6 Supplementary Note 6: More results of the *Petunia* data set S14

Figure S9. For Open-pFind and MODplus, unidentified PTM combinations can also be identified when the other PTM is pre-specified as variable modification . . . S14

7 Supplementary Note 7: Extra LSCC cohorts S15

Figure S10. Search results of the LSCC2 and LSCC3 data using PIPI-C at a peptide-level FDR < 0.01. (A) The number and proportion of UPSPs and PSMs identified from the LSCC1 data that are also identified in LSCC2 and LSCC3. (B) Five examples of conserved UPSPs with two PTMs identified from LSCC1, LSCC2, and LSCC3. (C) An example of a comparison of real MS2 and simulated MS2. The real MS2 indeed contains the backbone ions with relatively high intensities. S16

Figure S11. The number and proportion of UPSPs and PSMs identified from the COAD1 data that are also identified in COAD2. . . . . S18

8 Supplementary Note 8: Extra COAD cohort S18

1 Supplementary Note 1: Generation of simulated data sets

We used AlphaPeptDeep[1] to generate simulated MS2 spectra with selected PTMs as shown in **Table S1**. We allowed up to four PTMs in one peptide. Other parameters include a maximum missed cleavage site of 2. Since AlphaPeptDeep only predicted b and y ions, we manually added noise peaks to generate full MS2 spectra. The signal-to-noise ratio (SNR) is controlled by

$$\mathrm{SNR} = \sum I_s^2 / \sum I_n^2, \tag{S1}$$

where subscript s and n denote signal and noise, respectively. We obtained seven data sets from 881 template peptide sequences with different average SNRs ranging from 2.023 to 0.79. This SNR range was set based on the literature referenced in the literature[2–4].

Table S1: Parameters, PTMs, and sites used in AlphaPeptDeep for simulated data sets

<table>
<tr><td>Enzyme</td><td>trypsin</td><td rowspan="13">Variable modifications</td><td>Oxidation@G</td></tr>
<tr><td>Instrument</td><td>Lumos</td><td>Carboxyethyl@H</td></tr>
<tr><td>Max variable modifications</td><td>4</td><td>Carbonyl@L</td></tr>
<tr><td>Max missed cleavages</td><td>2</td><td>Oxidation@M</td></tr>
<tr><td>Min precursor charge</td><td>2</td><td>Deamidated@N</td></tr>
<tr><td>Max precursor charge</td><td>4</td><td>Dioxidation@P</td></tr>
<tr><td>Min peptide length</td><td>8</td><td>Deoxyhypusine@Q</td></tr>
<tr><td>Max peptide length</td><td>25</td><td>Ethanolyl@R</td></tr>
<tr><td>Fix modification</td><td>Carbamidomethyl@C</td><td>Malonyl@S</td></tr>
<tr><td rowspan="4">Variable modifications</td><td>Fluoro@A</td><td>Methylamine@T</td></tr>
<tr><td>Sulfide@D</td><td>Carbonyl@V</td></tr>
<tr><td>Decarboxylation@E</td><td>Chlorination@W</td></tr>
<tr><td>Nitro@F</td><td>Phospho@Y</td></tr>
</table>

2 Supplementary Note 2: Data preprocessing and tag extraction

The raw file is first converted into mgf format using ProteomeWizard MSConvert[5], then decharged and deisotoped using MS2-Deisotoper[6]. For each MS2 spectrum, the entire m/z range is divided into subranges of 100 Daltons, and the intensities of peaks are normalized by dividing them by the highest intensities in the corresponding subranges.

A weighted directed graph G is then constructed for tag extraction. The m/z difference between any two peaks is calculated to match potential amino acids. When an amino acid is matched, two nodes, n_i weighted by I_i (intensity of peak i), n_j weighted by I_j (intensity of peak j), and a directed edge $e_{i,j}$ weighted by $I_i + I_j$ are added to G. All paths from nodes with a zero in-degree to nodes with a zero out-degree are extracted as tags using depth-first search and ranked by the sum of the weights of nodes involved. All tags longer than the minimum tag length defined in the parameter file are collected.

3 Supplementary Note 3: Software and parameters used in the experiments

The version of all software used in this work is shown in **Table S2**. The common parameters used for the search engines in all the experiments are recorded in **Table S3**. All full parameter files are deposited on Zenodo at https://doi.org/10.5281/zenodo.14885715.

Table S2: Versions of software programs used in this work

Open-pFind[7]	3.2.0
MODplus[8]	2.01
Gurobi (Academic license)[9]	10.0.3
MSConvertGUI[5]	3.0.22238
MS2-Deisotoper[6]	N/A
MEME Suite[10]	5.5.5
Mascot[11]	2.7.0

Table S3: Common parameters used in the experiments. A single asterisk (*) denotes the search engine incorporating the corresponding variable modification setting. Double asterisk (**) denotes the search engine with both Phe→Cys@F and Unknown:248@N-term as variable modifications.

<table>
<tr><th>Exp</th><th>Software</th><th>MS1 tolerance</th><th>MS2 tolerance</th><th>Max cleavage sites</th><th>Fixed mod</th><th>Variable mod</th><th>Enzyme</th></tr>
<tr><td rowspan="3">1</td><td>PIPI-C</td><td rowspan="24">10ppm</td><td rowspan="9">0.01 Da</td><td rowspan="2">2</td><td rowspan="17">Carbamidomethyl@C</td><td rowspan="9">Oxidation@M</td><td rowspan="24">Trypsin</td></tr>
<tr><td>Open-pFind</td></tr>
<tr><td>MODplus</td><td>N/A</td></tr>
<tr><td rowspan="3">2</td><td>PIPI-C</td><td rowspan="2">1</td></tr>
<tr><td>Open-pFind</td></tr>
<tr><td>MODplus</td><td>N/A</td></tr>
<tr><td rowspan="3">3</td><td>PIPI-C</td><td rowspan="2">1</td></tr>
<tr><td>Open-pFind</td></tr>
<tr><td>MODplus</td><td>N/A</td></tr>
<tr><td rowspan="8">4</td><td>PIPI-C</td><td rowspan="8">0.02 Da</td><td rowspan="2">2</td><td rowspan="3">Oxidation@M
Acetyl@Prot-NTerm</td></tr>
<tr><td>Open-pFind</td></tr>
<tr><td>MODplus</td><td>N/A</td></tr>
<tr><td>Open-pFind*</td><td>2</td><td rowspan="3">Oxidation@M
Acetyl@Prot-Nterm,
one undetected PTM</td></tr>
<tr><td rowspan="2">MODplus*</td><td></td></tr>
<tr><td>N/A</td></tr>
<tr><td>Mascot</td><td rowspan="9">2</td><td>Oxidation@M
Acetyl@Prot-Nterm,
one undetected PTM,
GG@K</td></tr>
<tr><td>Open-pFind**</td><td>Oxidation@M
Acetyl@Prot-Nterm,
one undetected PTM,
Phe->Cys@F,
Unknown248@AnyNterm</td></tr>
<tr><td>LSCC1</td><td>PIPI-C</td><td>0.01 Da</td><td rowspan="5">Carbamidomethyl@C
TMT@K</td><td rowspan="5">TMT@Pep-N-term,
TMT@S</td></tr>
<tr><td>LSCC2</td><td>PIPI-C</td><td>0.02 Da</td></tr>
<tr><td>LSCC3</td><td>PIPI-C</td><td>0.02 Da</td></tr>
<tr><td>COAD1</td><td>PIPI-C</td><td>0.01 Da</td></tr>
<tr><td>COAD2</td><td>PIPI-C</td><td>0.02 Da</td></tr>
<tr><td>GBM1</td><td>PIPI-C</td><td>0.02 Da</td><td rowspan="2">Carbamidomethyl@C</td><td rowspan="2">Oxidation@M</td></tr>
<tr><td>GBM2</td><td>PIPI-C</td><td>0.02 Da</td></tr>
</table>

4 Supplementary Note 4: Details of the 21 synthetic data sets and the results

The details of all the 21 synthetic data sets[12] including the one used in experiment 2 are collected in Table S4. All the results are shown in **Figure S1**, **Figure S2**, and **Figure S3**.

Table S4: Information of the 21 synthetic data sets. Each data set contains tens of thousands of MS2 spectra with up to one PTM.

ID	Residue	Modification	Monoisotopic mass	Number of MS2
1	Lysine	Formylation	27.995	51861
2	Lysine	Acetylation	42.010	50511
3	Tyrosine	Phosphorylation	79.966	55131
4	Lysine	Methylation	14.016	49086
5	Lysine	Biotinylation	226.078	43572
6	Lysine	Butyrylation	70.042	48573
7	Lysine	Crotonylation	68.026	49398
8	Lysine	Dimethylation	28.031	47914
9	Lysine	Malonylation	86.000	50877
10	Lysine	Succinylation	100.016	49896
11	Proline	Hydroxyproline	15.995	46893
12	Lysine	Glutarylation	114.032	49197
13	Lysine	GlyGlycylation	114.043	47664
14	Lysine	Hydroxyisobutylation	86.037	49329
15	Lysine	Propionylation	56.026	49305
16	Lysine	Trimetylation	42.047	49296
17	Arginine	Citrullination	0.984	50087
18	Arginine	Dimethylation_asym	28.031	45183
19	Arginine	Dimethylation_symm	28.031	45666
20	Arginine	Methylation	14.016	45396
21	Tyrosine	Nitrotyrosine	44.985	48705

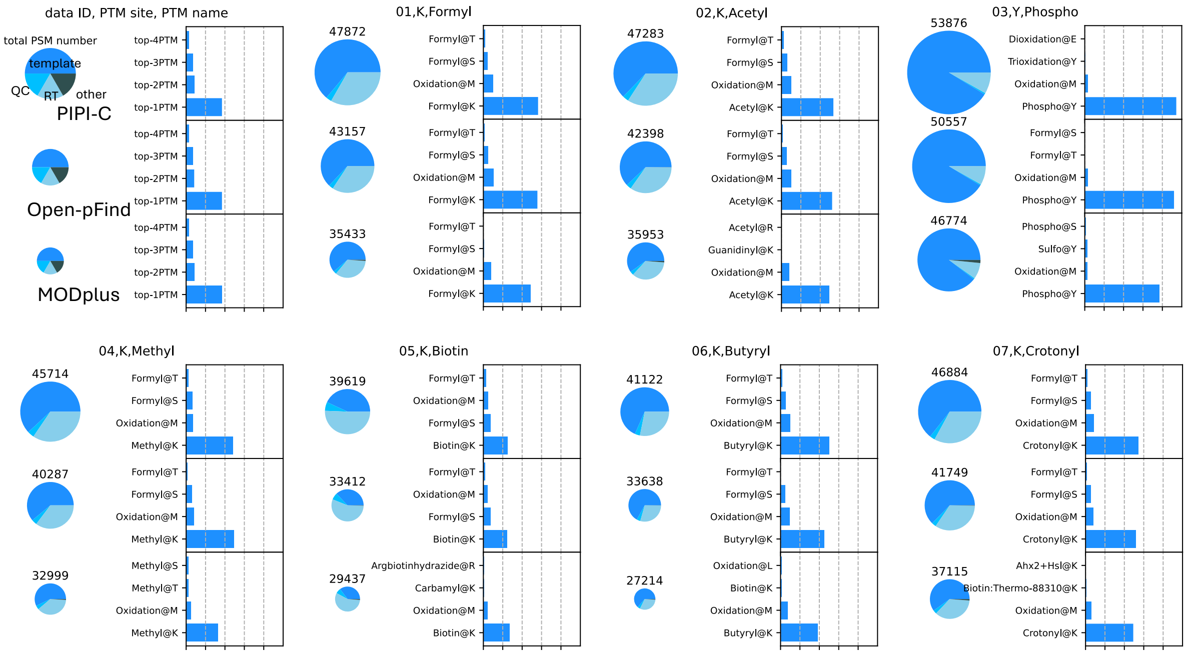

Figure S1: Comparison results of the synthetic data sets 01 to 07 at FDR of 0.01: bar chart, PSM numbers; pie chart, PTM numbers.

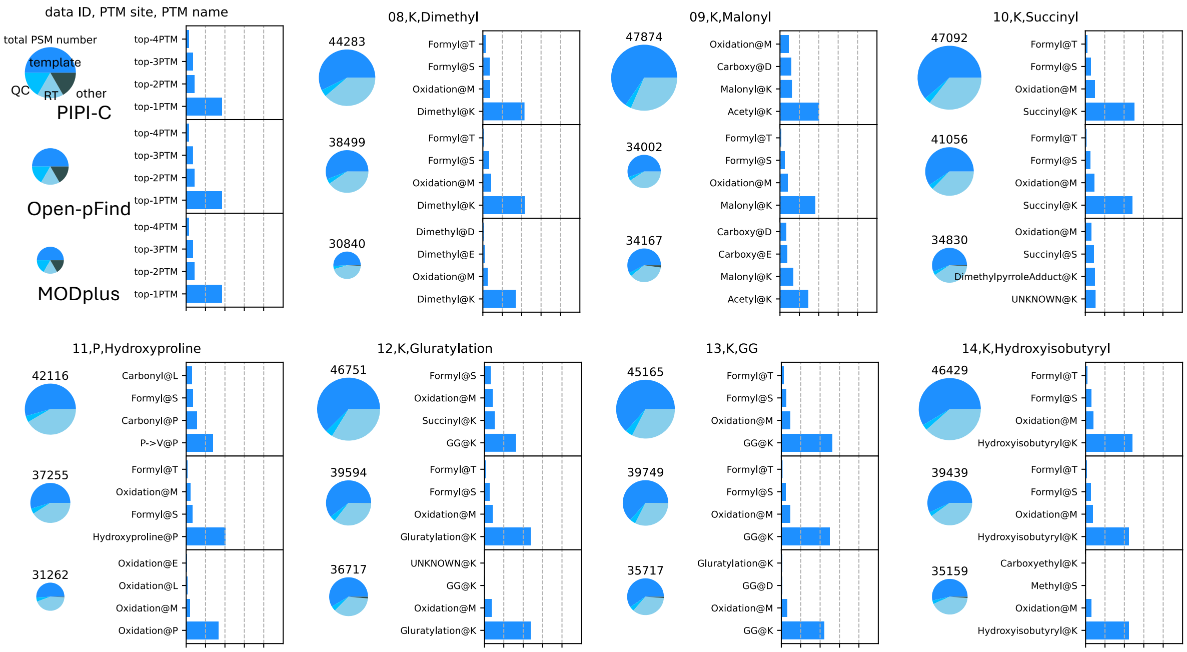

Figure S2: Comparison results of the synthetic data sets 08 to 14 at FDR of 0.01: bar chart, PSM numbers; pie chart, PTM numbers.

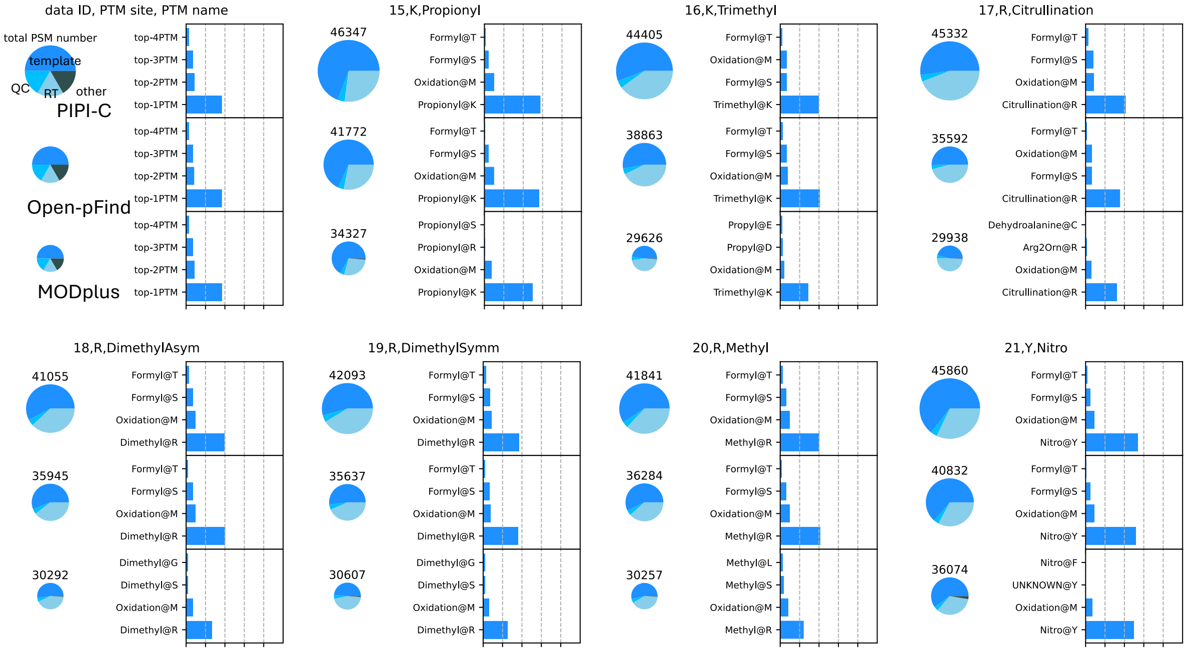

Figure S3: Comparison results of the synthetic data sets 15 to 21 at FDR of 0.01: bar chart, PSM numbers; pie chart, PTM numbers.

5 Supplementary Note 5: Results of replicated soybean data set

We repeated experiment 3 on five other replicated soybean data sets. The analysis is the same. As shown in **Figure** S4 to **Figure** S8, PIPI-C consistently outperform Open-pFind and MODplus in these replicate except R02.

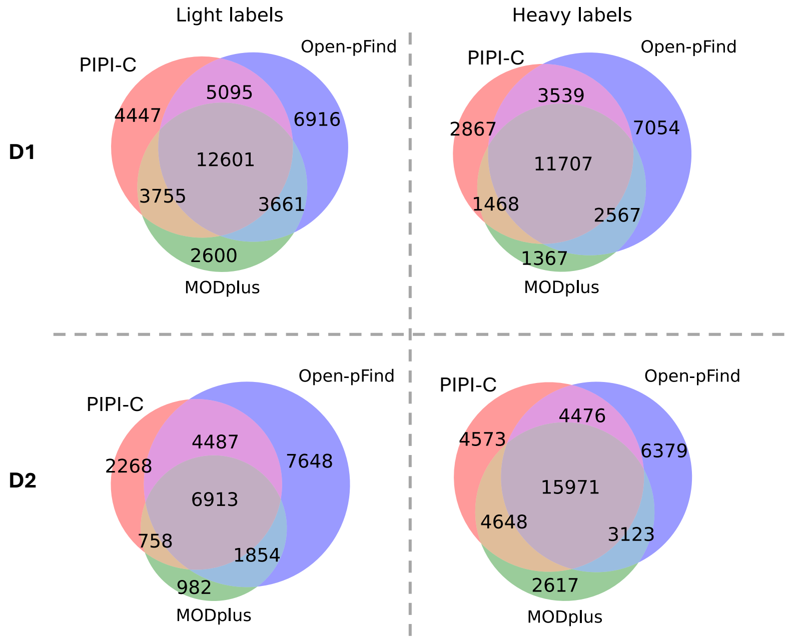

Figure S4: Replicate R02. Intersections of PSMs with fully dimethyl-labeled peptides identified from the soybean data sets. D1, D2: two samples in the data set.

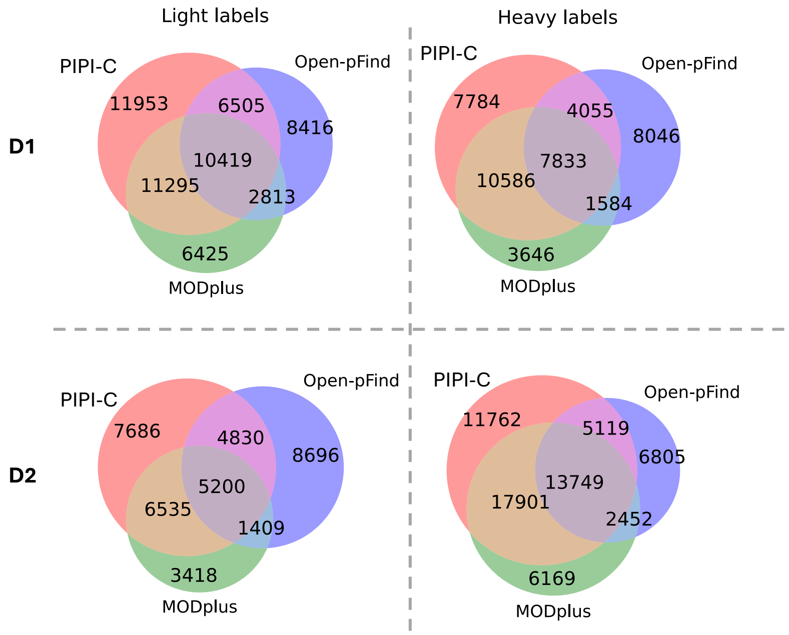

Figure S5: Replicate R03. Intersections of PSMs with fully dimethyl-labeled peptides identified from the soybean data sets. D1, D2: two samples in the data set.

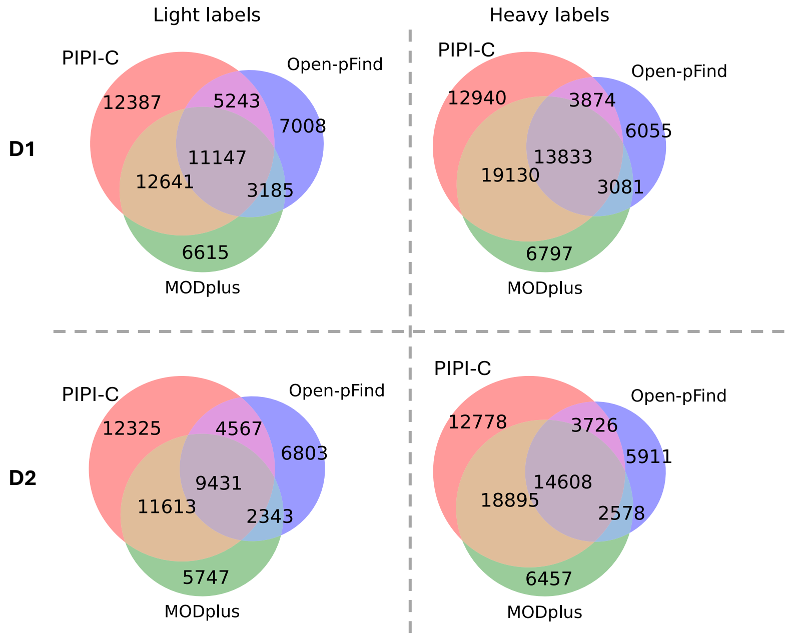

Figure S6: Replicate R04. Intersections of PSMs with fully dimethyl-labeled peptides identified from the soybean data sets. D1, D2: two samples in the data set.

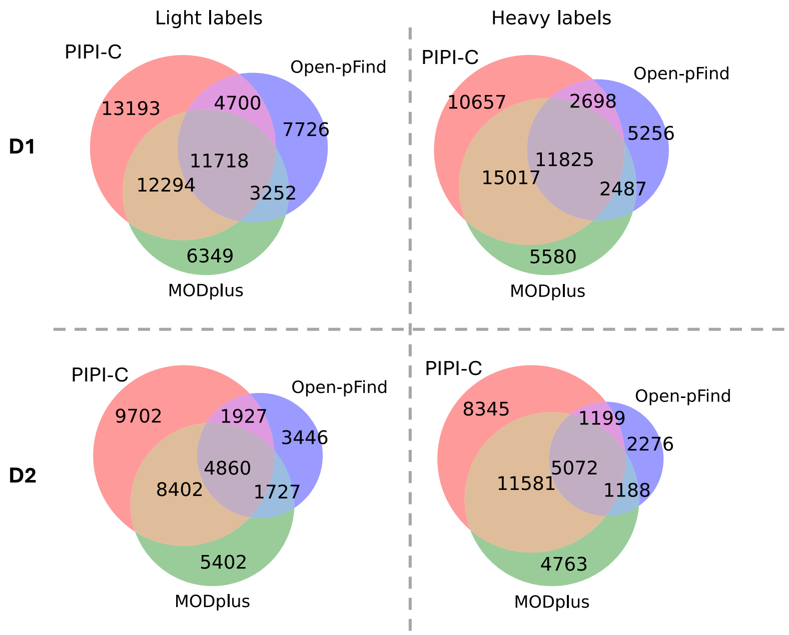

Figure S7: Replicate R05. Intersections of PSMs with fully dimethyl-labeled peptides identified from the soybean data sets. D1, D2: two samples in the data set.

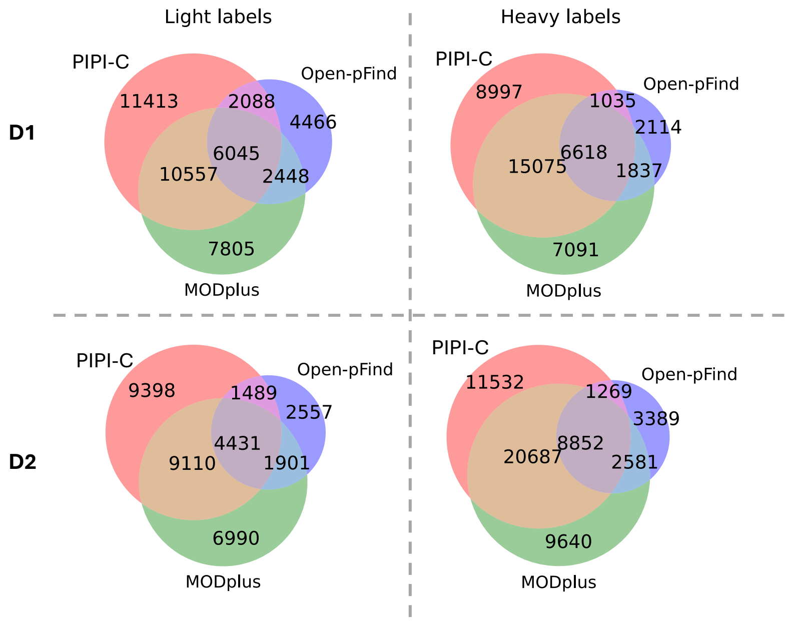

Figure S8: Replicate R06. Intersections of PSMs with fully dimethyl-labeled peptides identified from the soybean data sets. D1, D2: two samples in the data set.

6 Supplementary Note 6: More results of the *Petunia* data set

We recorded more results of the unidentified PTM combinations that can be identified when the PTM (other than GG@K) is pre-specified as a variable modification.

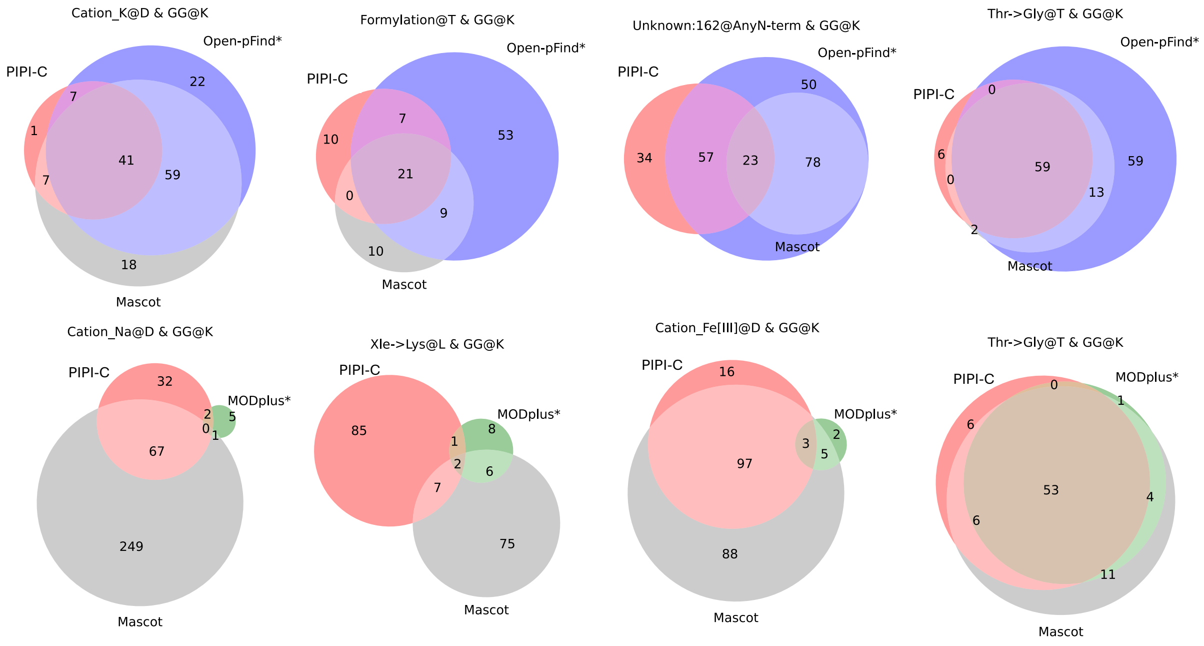

Figure S9: For Open-pFind and MODplus, unidentified PTM combinations can also be identified when the other PTM is pre-specified as variable modification

7 Supplementary Note 7: Extra LSCC cohorts

To verify the findings in the LSCC1 data, we used two extra LSCC cohorts (LSCC2, LSCC3) from different research groups as an independent validation. The first cohort [13], LSCC2, comprises 11,419,378 MS2 spectra collected from tumor tissues of 108 treatment-naive LSSC western patients. The data is organized into 29 batches (12 fractions in each batch) and labeled with TMT-6. The second cohort [14], LSCC3, comprises 3,147,669 MS2 spectra collected from tumor tissues of 25 treatment-naive Chinese patients with LSSC. The data is organized into five batches (20 fractions in each batch) and labeled with TMT-6. The search parameters of both cohorts are recorded in **Supplementary Table S3**.

At a peptide-level FDR of 0.01, we identified 1,333,749 peptides with TMT labels from LSCC2, among which 560,334 carried PTM(s) other than the TMT labels; these two numbers for LSCC3 are 1,248,897 and 604,987, respectively. The modified peptides from LSCC1 can be validated by LSCC2 and LSCC3 data, as shown in **Fig. S10A**. The first column is the number of PTMs in the peptides identified in LSCC1. The third to sixth columns are the total number of these peptides, and those that are also identified in LSCC2, in LSCC3, and in both LSCC2 and LSCC3. The color bars in the cells represent their proportion in the total number of peptides (the third column). The results from LSCC1 can be supported by LSCC2 and LSCC3 data in two aspects. First, the rows of "UPSP" show that among UPSPs with one PTM, about 27% and 45% are identified in LSCC2 and LSCC3, respectively, and about 20% are identified in both. Second, since some UPSPs are identified in multiple PSMs, we show the proportion of PSMs that are also identified by LSCC2 and LSCC3. Concretely, about 40% and 56% of PSMs with one PTM identified in LSCC1 are identified in LSCC2 and LSCC3, respectively. About 32% can be identified in both. While the proportions in the categories of with three or four PTMs are lower, it still shows good overlap (about 20%) for the two-PTM row.

Moreover, among the 860 upregulated UPSPs identified in LSCC1, 439 involve peptides with two PTMs, and 74 of them are also identified in both LSCC2 and LSCC3. We exemplified five of these

A

# PTM	UPSP/PSM	Total # in LSCC1	# by LSCC2	# by LSCC3	# by LSCC2 & LSCC3
1	UPSP	9311	2542	4156	1877
	PSM	418681	169717	234752	134956
2	UPSP	4456	886	949	476
	PSM	165632	50911	47490	30470
3	UPSP	108	16	15	8
	PSM	4399	1114	1206	857
4	UPSP	29	2	1	1
	PSM	1192	55	33	33

B

VADALTNAVAHVDD(248.199)MPNALSALSDLHAHKLR(-57.033)

FFESFGDLSTPD(17.027)AVM(15.995)GNPK

KV(15.995)LGA(85.064)FSDGLAHLDNLK

TYFPHFD(70.089)LSHGSAQ(159.068)VK

KVLGA(115.042)FSD(-13.979)GLAHLDNLK

C

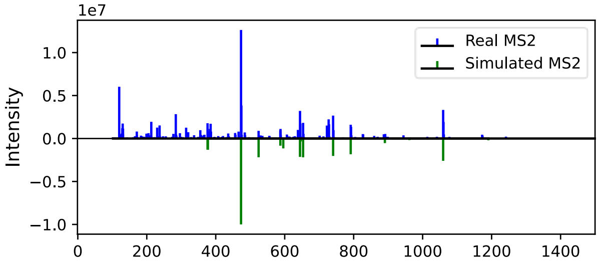

Figure S10: Search results of the LSCC2 and LSCC3 data using PIPI-C at a peptide-level FDR < 0.01. (A) The number and proportion of UPSPs and PSMs identified from the LSCC1 data that are also identified in LSCC2 and LSCC3. (B) Five examples of conserved UPSPs with two PTMs identified from LSCC1, LSCC2, and LSCC3. (C) An example of a comparison of real MS2 and simulated MS2. The real MS2 indeed contains the backbone ions with relatively high intensities.

conserved UPSP in **Fig. S10B**. These UPSPs correspond to 33 GO terms in total. 14 of these GO terms are frequently matched (more than 50 of these UPSPs per GO term), including protein binding, blood microparticle, cellular oxidant detoxification, oxygen binding, etc. In **Fig. S10C**, we simulated an MS2 that matched the second UPSP in **Fig. S10B** (F(229.163)FESFGDLSTPD(17.027)-AVM(15.995)GNPK), and compared it to one of the corresponding real MS2 (scan number 14383, fraction 13, pool 18). From this figure, we can see that the real MS2 indeed contains the backbone ions with relatively high intensities.

# PTM	UPSP/PSM	Total # in COAD1	# by COAD2
1	UPSP	505	252
	PSM	125702	68042
2	UPSP	241	85
	PSM	36934	15676
3	UPSP	21	8
	PSM	4590	2422
4	UPSP	3	1
	PSM	304	14

Figure S11: The number and proportion of UPSPs and PSMs identified from the COAD1 data that are also identified in COAD2.

8 Supplementary Note 8: Extra COAD cohort

To check the findings in the COAD1 data, we used an extra COAD cohort (COAD2) from a different research group[15] as an independent validation. COAD2 comprises 12,618,939 MS2 spectra collected from tumors and paired distant normal tissues from 104 COAD patients. The data is labeled with TMT-10. The search parameters for this cohort are recorded in **Supplementary Table S3**.

At a peptide-level FDR of 0.01, we identified 3,182,698 peptides with TMT labels from COAD2, among which 1,174,681 carried PTM(s) other than the TMT labels. As shown in **Fig. S11**, the first column is the number of PTMs in the peptides identified in COAD1, the third column is the total number of these peptides, and the fourth column is the number of those that are also identified in COAD2. The color bars in the cell represent their proportion in the total number of peptides (the third column). The results from COAD1 can be supported by COAD2 in two aspects. First, the rows of "UPSP" show that among UPSPs with one PTM, about 50% are identified in COAD2. Second, since some UPSPs are identified in multiple PSMs, we show the proportion of PSMs that are also identified by COAD2, i.e., about 54% of PSMs with one PTM identified in COAD1 are identified in COAD2. The proportions of PSMs with two to four PTMs identified by COAD2 are about 42%, 53%, and 5%, respectively.

References

[1] Zeng, W. *et al.* AlphaPeptDeep: A modular deep learning framework to predict peptide properties for proteomics. *Nature Communications* **13**, 7238 (2022).

[2] Michalski, A., Cox, J. & Mann, M. More than 100,000 detectable peptide species elute in single shotgun proteomics runs but the majority is inaccessible to data-dependent LC-MS/MS. *Journal of Proteome Research* **10**, 1785–1793 (2011).

[3] Liu, K., Li, S., Wang, L., Ye, Y. & Tang, H. Full-spectrum prediction of peptides tandem mass spectra using deep neural network. *Analytical Chemistry* **92**, 4275–4283 (2020).

[4] Lai, S., Zhao, P., Zhou, C., Li, N. & Yu, W. PIPI2: Sensitive tag-based database search to identify peptides with multiple post-translational modifications. *Journal of Proteome Research* **23**, 1960–1969 (2024).

[5] Chambers, M. C. *et al.* A cross-platform toolkit for mass spectrometry and proteomics. *Nature Biotechnology* **30**, 918–920 (2012).

[6] Tay, A. P., Liang, A., Hamey, J. J., Hart-Smith, G. & Wilkins, M. R. MS2-Deisotoper: A tool for deisotoping high-resolution MS/MS spectra in normal and heavy isotope-labelled samples. *Proteomics* **19**, 1800444 (2019).

[7] Chi, H. *et al.* Comprehensive identification of peptides in tandem mass spectra using an efficient open search engine. *Nature Biotechnology* **36**, 1059–1061 (2018).

[8] Na, S., Kim, J. & Paek, E. MODplus: Robust and unrestrictive identification of post-translational modifications using mass spectrometry. *Analytical Chemistry* **91**, 11324–11333 (2019).

[9] Gurobi Optimization, LLC. Gurobi Optimizer Reference Manual (2023). URL https://www.gurobi.com.

[10] Bailey, T. L., Johnson, J., Grant, C. E. & Noble, W. S. The MEME suite. *Nucleic Acids Research* **43**, W39–W49 (2015).

[11] Perkins, D. N., Pappin, D. J., Creasy, D. M. & Cottrell, J. S. Probability-based protein identification by searching sequence databases using mass spectrometry data. *ELECTROPHORESIS: An International Journal* **20**, 3551–3567 (1999).

[12] Zolg, D. P. *et al.* ProteomeTools: Systematic characterization of 21 post-translational protein modifications by liquid chromatography tandem mass spectrometry (LC-MS/MS) using synthetic peptides. *Molecular & Cellular Proteomics* **17**, 1850–1863 (2018).

[13] Stewart, P. A. *et al.* Proteogenomic landscape of squamous cell lung cancer. *Nature Communications* **10**, 3578 (2019).

[14] Pan, L. *et al.* Proteomic and phosphoproteomic maps of lung squamous cell carcinoma from Chinese patients. *Frontiers in Oncology* **10**, 963 (2020).

[15] Wang, X. *et al.* Age-, sex-and proximal–distal-resolved multi-omics identifies regulators of intestinal aging in non-human primates. *Nature Aging* **4**, 414–433 (2024).
